# Supplementary material for: Systematic investigation of the deformation mechanisms of a γ-TiAl single crystal
Source: Sci Rep. 2018 Oct 12;8:15200. doi: 10.1038/s41598-018-33377-z (PMC6185918; doi:10.1038/s41598-018-33377-z)
Supplement: Supplementary file 1 — Supplementary Information [file 41598_2018_33377_MOESM1_ESM.pdf]

Supplementary Information for

Systematic investigation of the deformation mechanisms of a γ-TiAl single crystal

Byungkwan Jeong[a], Jaemin Kim[a], Taegu Lee[a], Seong-Woong Kim[b,**], Seunghwa Ryu[a,*]

[a] Department of Mechanical Engineering & KI for the Nano Century, Korea Advanced Institute of Science and Technology, Daejeon 34141, Republic of Korea

[b] Titanium Department, Korea Institute of Materials Science, Changwon 51508, Republic of Korea

[*] Corresponding author. Tel.: +82-42-350-3019; fax: +82-42-350-3059

Email address: ryush@kaist.ac.kr (Seunghwa Ryu)

[**] Co-corresponding author. Tel.: +82-55-280-3000; fax: +82-55-280-3333

Email address: mrbass@kims.re.kr (Seong-Woong Kim)

Supplementary Note 1. The ideal critical resolved shear stress to emit a twinning partial.

Twinning partial involves stacking fault energy unlike full dislocation. Therefore, in order to analyze the twinning partial emission, we must consider the effect of the stacking fault energy. In order to calculate the ICRSS of a twinning partial, we need to consider Peierls stress, the interaction force between leading and trailing partial and stacking fault energy. Therefore, the critical resolved shear stress to emit a twinning partial is given as [1],

$$\tau_{tp} = \frac{\mu_{tp} b_{tp}}{d} + \frac{\gamma_{SISF} - F_{12}}{b_{tp}} + \tau_p \qquad (1)$$

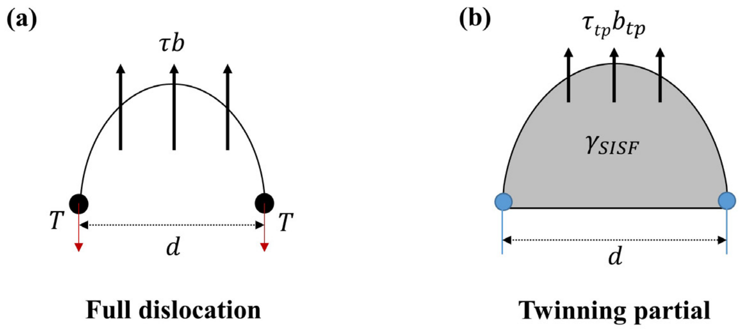

Figure S1. Comparison of the ideal critical resolved shear stress of full dislocation and twinning partial.

where τ_{tp} is the ideal critical resolved shear stress to emit a twinning partial, γ_{SISF} is the stacking fault energy, d is the sample size, b_{tp} is the magnitude of the Burgers vector of the twinning partial, μ_{tp} is the effective shear modulus for the twinning partial, F_{12} is the interaction force between the leading and trailing partial, and τ_p is the Peierls stress. The lattice friction stress (the Peierls stress) at 0K is estimated by a DFT method to be small value of around 0.01C44 (C44=68 GPa) [2]. The lattice friction stress at 300K is significantly smaller due to thermal fluctuation as shown earlier studies (Kang et al., 2014). Furthermore, there is no interaction between leading and trailing partial when twinning is observed, and we can approximately simplify (1) as follows.

$$\tau_{tp} \approx \frac{\mu_{tp} b_{tp}}{d} + \frac{\gamma_{SISF}}{b_{tp}} \qquad (2)$$

Supplementary Note 2. Calculation of the effective shear modulus

To calculate the effective shear modulus, we first calculated elastic constants for γ-TiAl single crystal using Farkas & Jones EAM potential and using first principle calculation. Following the method of Scattergood and Bacon [3], the effective shear modulus can be calculated as follows:

$$\mu = \frac{4\pi}{b^2} E_s \qquad (6)$$

where E_s is the pre-logarithmic energy factor of screw dislocations. All the results of the elastic constants and the effective shear modulus can be found in Table 1.

Supplementary Figure

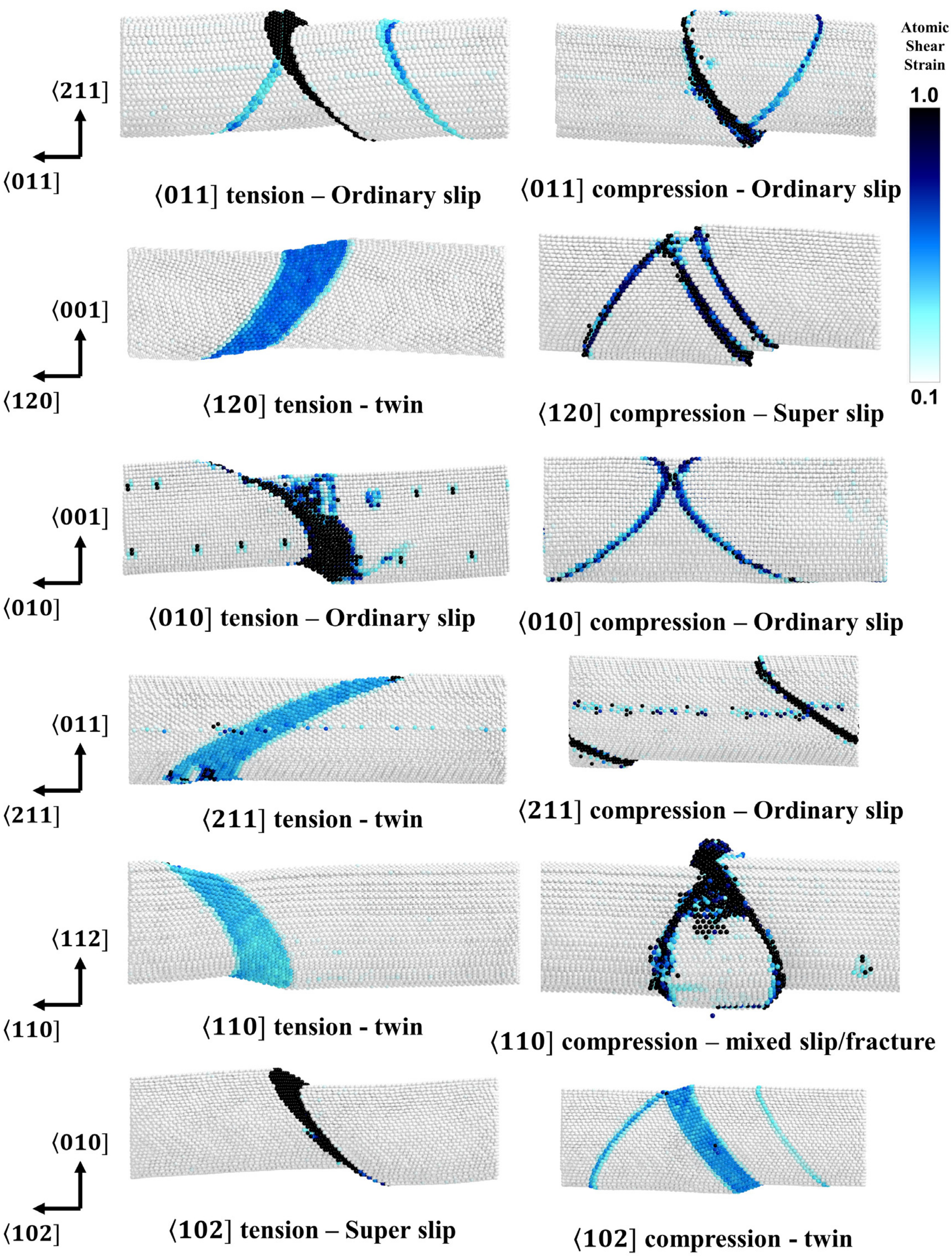

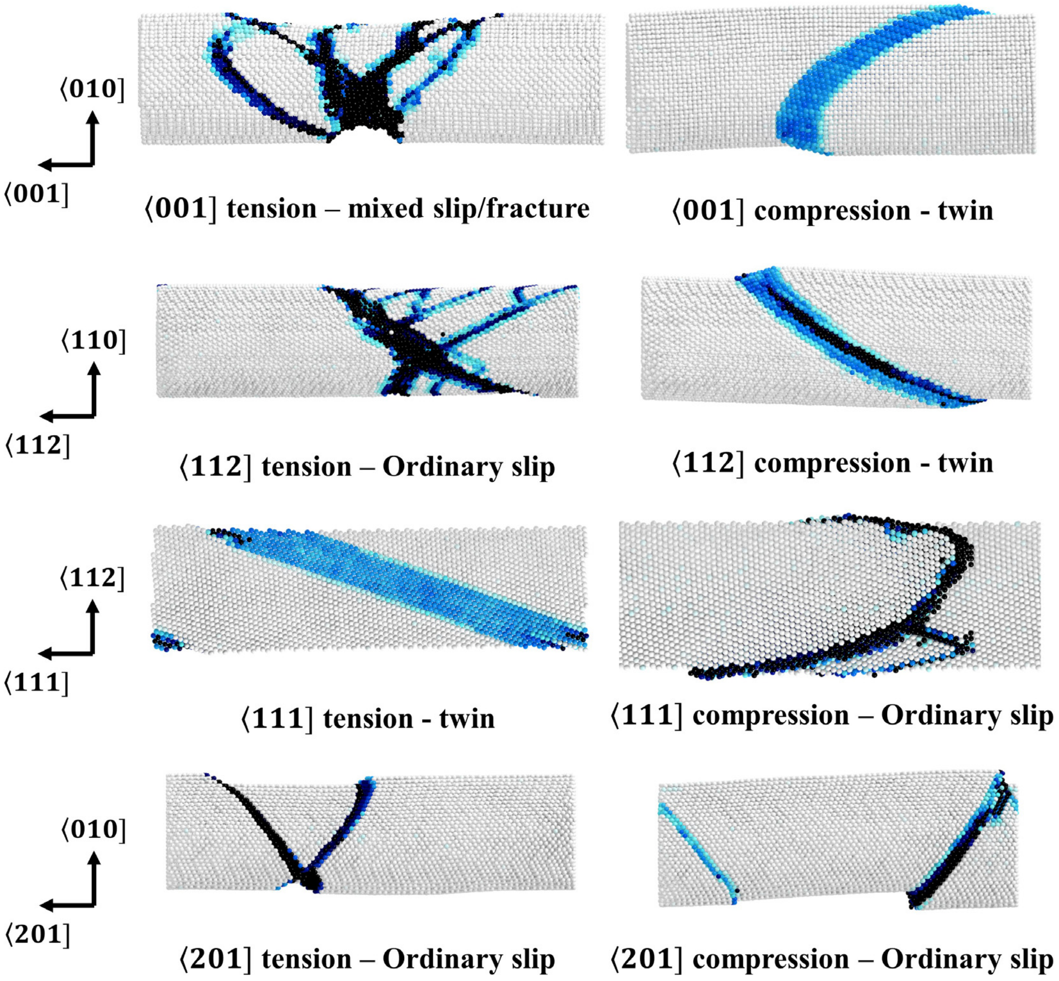

Fig. S2. Simulation results for 10 orientations under uniaxial tension/compression.

Fig. S2 shows deformation results for 10 different orientations under uniaxial tension or compression. For each orientation, the observed deformation modes are also indicated.

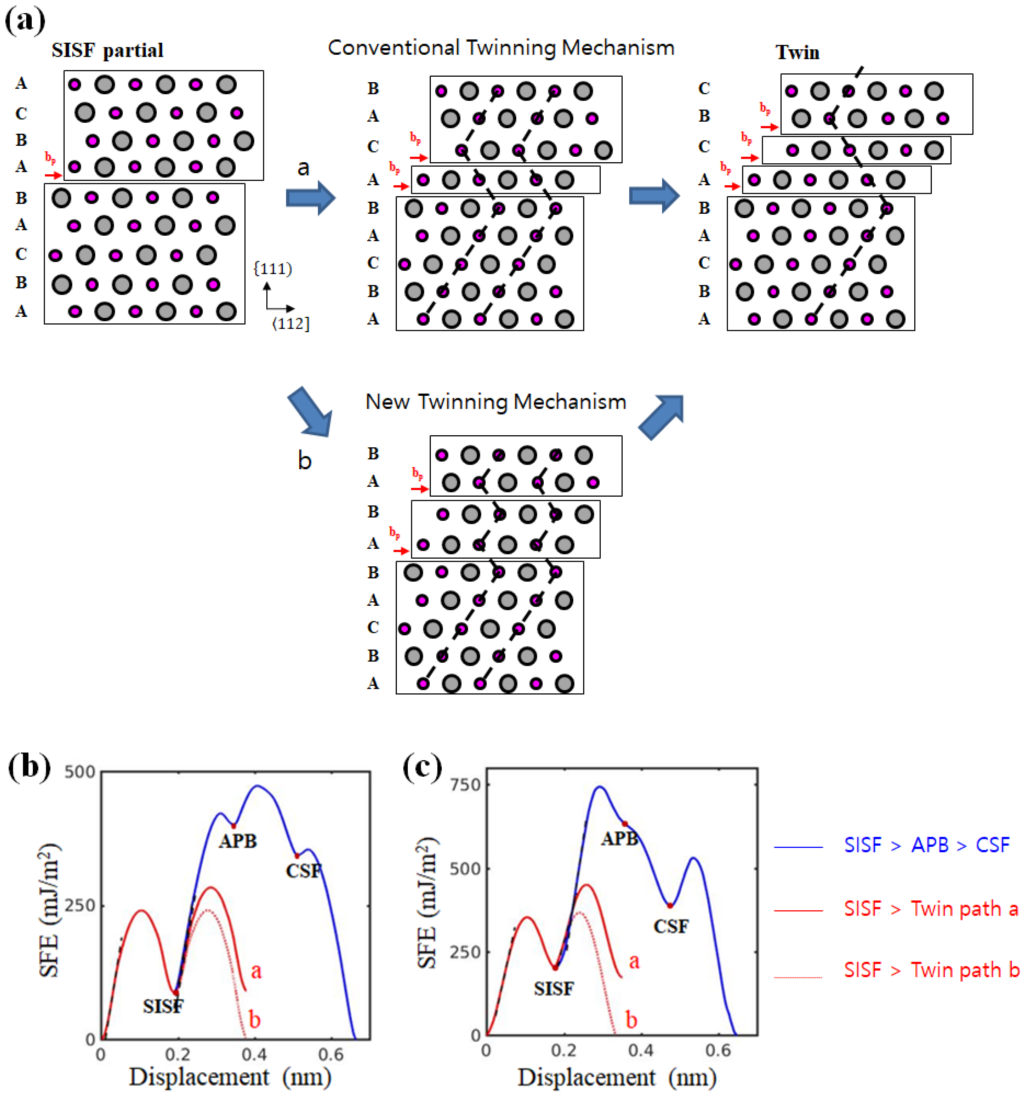

Fig. S3. (a) Schematic of conventional and new twinning path reported by Wang et al. [5] (b) Stacking fault energy curve obtained from Farkas EAM potential (c) Stacking fault energy curve obtained from DFT calculation

Fig. S3 shows the schematic of conventional and new twinning path reported by Wang et al.[5], as well as the generalized stacking fault energy curves obtained by Farkas EAM potential and DFT calculations. For both calculations (Fig S3(b)-(c)), it is evident that the new twinning path has lower ideal critical resolved shear stress (ICRSS) than the conventional path does. Still, whichever twining path is considered, twinning is predicted to be the preferred deformation mechanism along [001] and [$\bar{2}$33] compressions, while experiments show that samples under these two loading conditions are deformed by superdislocations.

Supplementary Table S1. Critical stress of partial slips for the $[\bar{1}52]$ orientation in compression loading on {111) slip planes.

{111) slip planes	Step 1		Step 2		Prediction
	$\sigma^c{}_{CSF}$ (GPa)	$\sigma^c{}_{SISF}$ (GPa)	$\sigma^c{}_{o}$ (GPa)	$\sigma^c{}_{Isuper}$ (GPa)	
(111)	19.42	1073	10.83	INF	Ordinary slip
$(11\bar{1})$	35.15	INF	77.91	37.6	Inverse super slip
$(1\bar{1}1)$	20.15	INF	INF	15.3	Inverse super slip
$(\bar{1}11)$	17.91	INF	15.48	41.3	Ordinary slip

For $[\bar{1}52]$ orientation, which is deformed by super slip on the $(1\bar{1}1)$ slip plane in the experiment [4], we predicted that the preferred deformation mode is ordinary slip on the $(\bar{1}11)$ slip plane because it has the lowest $\sigma^c{}_{CSF}$. However, $\sigma^c{}_{CSF}$ on $(\bar{1}11)$ and $(1\bar{1}1)$ slip planes differ by approximately 11%, which can be caused by different Al composition or the limitation of the accuracy of the DFT calculation.

Reference

[1] K.P.D. Lagerlof, J. Castaing, P. Pirouz, A.H. Heuer, Nucleation and growth of deformation twins: a perspective based on the double-cross-slip mechanism of deformation twinning, Philos. Mag. A. 82 (2002) 2841–2854. doi:10.1080/01418610210157931.

[2] C. Woodward, S.I. Rao, Ab-initio simulation of (a/2)<110] screw dislocations in γ-TiAl, Philos. Mag. 84 (2004) 401–413. doi:10.1080/14786430310001611626.

[3] D.J. Bacon, D.M. Barnett, R.O. Scattergood, Anisotropic continuum theory of lattice defects, 23 (1979).

[4] H. Inui, M. Matsumuro, D.-H. Wu, M. Yamaguchi, Temperature dependence of yield stress, deformation mode and deformation structure in single crystals of TiAl (Ti−56 at.% Al), Philos. Mag. A. 75 (1997) 395–423. doi:10.1080/01418619708205149.

[5] L. Wang, P. Guan, J. Teng, P. Liu, D. Chen, W. Xie, D. Kong, S. Zhang, T. Zhu, Z. Zhang, E. Ma, M. Chen, X. Han, New twinning route in face-centered cubic nanocrystalline metals, Nat. Commun. 8 (2017) 2142. doi: 10.1038/s41467-017-02393-4.
